# The Role of Root Morphology and Pulling Direction in Pullout Resistance of Alfalfa Roots

**DOI:** 10.3389/fpls.2021.580825

**Published:** 2021-02-17

**Authors:** Qihong Yang, Chaobo Zhang, Pengchong Liu, Jing Jiang

**Affiliations:** ^1^Changjiang River Scientific Research Institute, Changjiang Water Resources Commission, Wuhan, China; ^2^College of Water Resources Science and Engineering, Taiyuan University of Technology, Taiyuan, China

**Keywords:** *Medicago sativa* L., root pullout resistance, pulling direction, mechanical effect, root and soil interaction

## Abstract

There is a growing consensus on soil conservation by mechanics of plant root system. In order to further study how root system exerts its mechanical properties during soil reinforcing process and which morphological indicator is suitable for reflecting pullout resistance, *in-situ* vertical pullout test (VPT) and 45° oblique pullout test (OPT) were performed on alfalfa (*Medicago sativa* L.) roots in the loess area. The results showed that the failure mode of alfalfa roots was pulling out in this study. The peak pullout resistance of the roots increased with root diameter, root length and root surface area, and power law relationships were observed between the pullout resistance and the morphological indices: root diameter, root length and root surface area. The maximum gray relational degree of the morphological indices was 0.841 (VPT) and 0.849 (OPT) for root surface area, suggesting that root surface area was a more significant root morphological index affecting root pullout resistance than root diameter and root length, and was more suitable for characterizing the difference in peak pullout resistance of roots with different size. The index could be used to validate the methods for predicting root pullout capacity. The value of peak pullout resistance was 17.2 ± 2.3 N in VPT test and 28.2 ± 3.8 N (mean ± SE) in OPT test, and a significant difference was observed between the two tests, which showed that the pulling direction significantly affected the peak pullout resistance of alfalfa roots. Vertical pullout test, giving the safety margin, was suggested to determine root pullout resistance for estimate of root reinforcement.

## Highlights

- Root surface area was a better morphological index for reflecting pullout resistance.
- Pulling direction significantly affected root pullout resistance.
- Vertical pulling was recommended for root reinforcement.

## Introduction

Due to the natural causes such as sparse vegetation, loose soil, topographic relief and strong precipitation, as well as man-made reasons such as overgrazing, mining, deforestation and wasteland, soil erosion is widely occurred in the world, such as the Loess Plateau, the most serious area of soil erosion in China (Zhao et al., 2013). Vegetation measure is an effective way to prevent soil erosion (Mu et al., 2019). In addition to the hydrological effects of plant surface parts, such as intercepting rainfall by leaves, retaining water and inhibiting surface runoff by litter (Mu et al., 2019; Yang et al., 2019, 2020), plant roots, as a media of communication between plants and soils, exert the mechanical effects including reinforcing and anchoring soil, and improving stability of slopes (Schwarz et al., 2010a; Simon and Collison, 2010; Castillo and Smith-Ramírez, 2018; Wang et al., 2019; Su et al., 2020). On the one hand, the root system in the slope can be regarded as a pre-stressed reinforced material, which produces frictional reinforcement on the soil and improves the soil cohesion (Waldron, 1977; Wu, 1979). In addition, the root system in the slope soil acts as a mesh wrap and limits the lateral deformation of the soil, which reduces the maximum shear stress on the soil. Shear strength of the soil is consequently improved (Schwarz et al., 2010b; Liang et al., 2017). On the other hand, the vertical thick roots of plants can extend to deeper stable soil layer. When the sliding plane into which roots are crossing is subject to the forces which tend to shear and either break or pull the roots out, the shear deformation and stress of soil can be transferred to deep stable soil via plant roots. Roots resist the forces, and mechanically reinforce the soil and increase slope stability (Mickovski et al., 2007). Therefore, root pullout resistance is an important parameter to characterize the mechanical action of plant roots.

The three kinds of tests, pullout test of the whole plant, also named uprooting test (Leung et al., 2018; Bau′ et al., 2019), pullout test on remolded soil-root samples (Mickovski et al., 2007; Schwarz et al., 2011; Giadrossich et al., 2013) and *in-situ* pullout test (Pollen, 2007; Vergani et al., 2016; Zhang et al., 2020) are often used to study the root pullout mechanical properties. Pullout test of the whole plant can determine the pullout resistance of the plant, but it is not able to get pullout resistance of single root. Remolded samples adopt carefully chosen root segments, which have a greatly different bonding with soil from the natural condition. It is difficult to control root age and physical parameters of the soil in the nature under *in*-*situ* pullout test. However, *in-situ* pullout test is carried out on undisturbed soil, in which the status of bonding and interweaving between roots and soil is natural in the raw before test. It should be more reliable to reflect the real interaction between single root and its surrounding soil than remolded soil pullout test. Besides, *in-situ* pullout test is the most important test to estimate root reinforcement by root bundle model (Schwarz et al., 2013).

Root pullout resistance is affected by a number of factors. Some are related to the soil in which roots are embedded, such as soil type and soil water content (Dupuy et al., 2005; Pollen, 2007), and others are related to the root geometries (branching patterns), root material property (e.g., stiffness, strength) and root morphology (e.g., diameter, length, surface area). Previous researches show pullout resistance of roots in sand may be greater than that in silty soil when soil water content is low, but smaller when soil water content is high (Schwarz et al., 2011). Soil water content decreases soil aggregate stiffness and friction (Schwarz et al., 2011), and sand is affected more seriously than silty soil. However, another view is that soil water content can increase soil matric potential and soil effective stress, and then increase root resistance (Mickovski et al., 2007). Stokes et al. (1996), Mickovski et al. (2007), and Kamchoom et al. (2014) have developed several simple and complicated branching patterns using model roots and studied their effects on root pullout resistance. The presence and position of branch roots strongly affect the peak pullout resistance. The deeper branch roots can result into greater pullout resistance (Bransby et al., 2006). Some studies show that pullout resistance of branched roots is increased 1.5–2 times as large as that of unbranched roots (Schwarz et al., 2011; Giadrossich et al., 2013). Root stiffness and strength have major effects on the root pullout behavior, which has been recognized that more rigid and stronger roots show greater pullout resistance capacity than more flexible and weaker roots (Mickovski et al., 2007). Pullout resistance of plant roots also increases with the increase of the three root morphological indices: root diameter, root length and surface area (Ennos, 1990; Mickovski et al., 2010). However, it is unknown exactly which root morphological index has more significant effect on root pullout resistance, and is more suitable for characterizing the difference of peak pullout resistance in different roots. The index is expected to be incorporated into the prediction of root pullout resistance and the evaluation of soil reinforcement. Besides, pullout resistance of roots adopted in root reinforcement model is often derived from the tests pulling roots vertically to soil surface (Schwarz et al., 2013). The effect of pulling roots in different directions on root peak pullout resistance is still undefined.

Therefore, the objectives of this study were (1) to analyze the effects of root diameter, root length and root surface area, and pulling direction on the pullout resistance of alfalfa roots by *in-situ* pullout tests, and (2) to search a suitable root morphological index for predicting peak pullout resistance of roots. The index is intended to provide a basis for the study of root pullout performance and root-soil mechanics.

## Materials and Methods

### Study Area

The experiment was carried out in 2017 at the College of Water Resources Science and Engineering, Taiyuan University of Technology, Taiyuan City, Shanxi Province, China. Taiyuan City (latitude 37°54′, east longitude 112°33′) is located in the north-central part of Shanxi Province, with the highest altitude of 2,670 m and the lowest point of 760 m. The average elevation is about 800 m. The annual average temperature is about 9.5°C, and the average frost-free period of 1 year is 202 days. The average annual rainfall is 456 mm. The climate is a typical continental climate with relatively dry air and less rainfall.

### Samples Preparation

**Figure 1:**
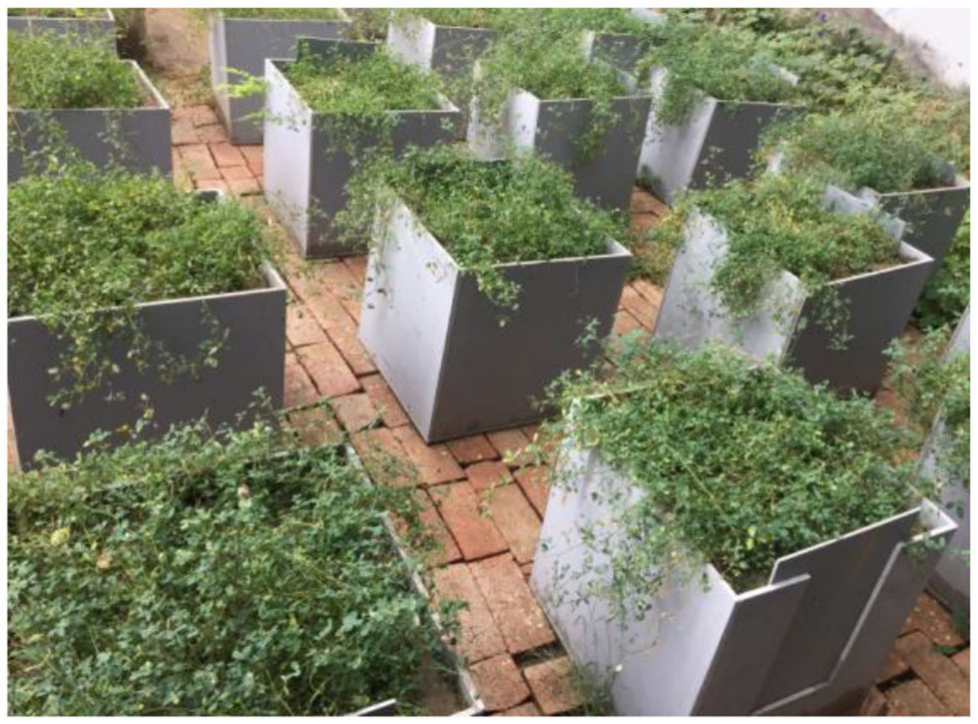
Cultivation of alfalfa plants.

The Loess Plateau, with an area of about 640,000 km^2^, is the main sediment source of the Yellow River of China. It is one of the regions with the most serious soil erosion and a leading vegetation restoration region in China and the world (Zhao et al., 2020). Alfalfa (*Medicago sativa* L.) is a representative species in the loess region of China. It can not only provide high-quality forage feed, having good economic benefits, but also can be used to effectively control soil erosion. Alfalfa used in this study is a kind of taproot plant having obvious and long and thick main root, and short and thin lateral roots. Therefore, loess and alfalfa were used in this study. Alfalfa was planted in 16 pots (Figure 1). The soil was sampled from the loess near Taiyuan, and then air-dried and sieved through a 2 mm sieve. In order to maintain the similarity of the bulk density and uniformity of the soil in the natural environment, the soil was sampled and filled in five layers, each layer 10 cm. The potted plants were kept in the natural environment with a growth period of 12 months. During the *in-situ* pullout tests, the average moisture content of the loess was 14.27% determined by dry oven method, and the bulk density of the loess was 1.25 g·cm^−3^ measured by central knife method.

### *In-situ* Pullout Test

**Figure 2:**
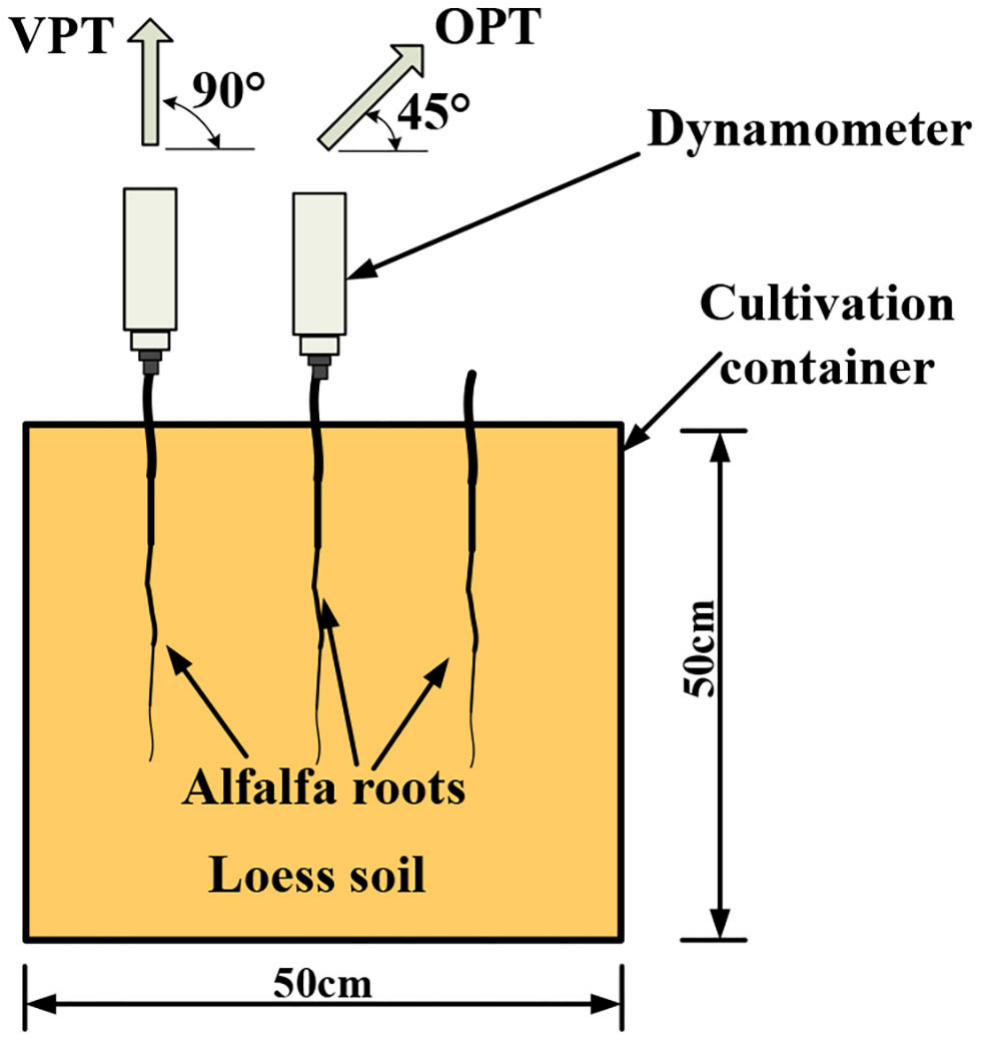
Vertical pullout test (VPT) and 45° oblique pullout test (OPT) of alfalfa roots.

Two frequently used *in-situ* pullout tests, vertical pullout test (VPT) and 45° oblique pullout test (OPT), were carried out (Figure 2). The main experimental steps of the *in-situ* pullout tests were as follows: (1) Fixing root. Root segment with a length of 3 cm was exposed to the air from the soil and fixed to the clamp of the dynamometer HANDPI NK-100 (HANDPI, Yueqing Handpi Instruments Co., Ltd.). (2) Measuring root diameter. Diameter of the exposed root segment was measured three times at a distance of 1 cm to the soil surface, and the average value was taken as the diameter (*D*, mm) of the root. (3) Measuring peak pullout resistance. The dynamometer was first zeroed and then moved evenly vertically or obliquely at approximate 100 mm·min^−1^ until the root was failed, just then the value of peak pull force (*F*, N) was recorded. (4) Measuring root length. The whole root length (*L*, mm) was measured with a tape. (5) Calculating root surface area. Considering the root system as a cone, root surface area (*S*, mm^2^) was calculated by the formula *S* = π*DL*/2. Because the size of root diameter cannot be repeated, it is not realistic to pull the roots with the same diameter to achieve the repetition. According to the usual method of previous researches, the root pullout tests were carried out by simply random sampling. In the sampling, the root diameter of the two groups of pulling angles was controlled in the same range, and the root diameter of single sample between the two groups was kept as close as possible. No significant difference of root diameter was existed between the two groups.

### Gray Relational Analysis

Gray relational analysis is proposed by Deng (1990) from gray systems methodology. It is an approach for quantitative analysis of dynamic process using similarity of trend and pattern between the reference sequence and comparative sequences. In the gray relational model, it hypothesizes that the closer relationship between reference sequence and comparative sequences, the more similar of trend and pattern of the dynamic processes. The comparative sequence, of which trend and rate is closer to the reference sequence, has stronger associations with the reference sequence (Li et al., 2017). The gray relational analysis method used in this study was as follows:

(1) Determine the reference sequence and the comparative sequences. The reference sequence *x*_0_ = {*x*_0_(1), *x*_0_(2),......, *x*_0_(n)} was determined to reflect the behavior characteristics of the dynamic system. Then, the comparative sequences *x*_*i*_ = {*x*_*i*_(1), *x*_*i*_(2),......, *x*_*i*_(n)}, and i = 1, 2,..., *m*, were constructed to represent the multiple factors in the dynamic system. The *m* was the total number of the comparative sequences *x*_*i*_.
(2) The dimensions or magnitudes of the above *i* + 1 sequences were different and needed to be dimensionless so that the evaluation results were comparable. Using the averaging method, each metric data for each sequence was non-dimensionalized:

(1) $$x_{i}^{\prime }(k)=\frac{x_{i}(k)}{\frac{1}{n}\sum_{k=1}^{n}x_{i}(k)}\;(k=1,2,...,n;\;i=1,2,...,m)$$

where $x_{i}^{\prime }(k)$ is the *k*-th value in the *i*-th sequence after dimensionless data values; *x*_*i*_(*k*) is the *k*-th value in the *i*-th raw sequence; *k* = 1, 2,..., *n* represent the value at the point in time *k*.

(3) For each comparative sequence, the gray relevancy coefficient ξ_i_ (*k*) represented the association extent between reference sequence *x*_0_ and comparative sequences *x*_*i*_ at a certain point in time *k*:

(2) $$\xi_{0i}(k)=\frac{\Delta_{\min}+\rho \Delta_{\max}}{\Delta_{0i}(k)+\rho \Delta_{\max}}$$

where $\Delta_{0i}(k)=|x_{0}^{\prime }(k)-x_{i}^{\prime }(k)|$; Δ_min_ is two-level minimum, $\Delta_{\min}=\underset{i}{\min}\;\underset{k}{\min}|x_{0}^{\prime }(k)-x_{i}^{\prime }(k)|$; Δ_min_ is two-level maximum, $\Delta_{\max}=\underset{i}{\max}\;\underset{k}{\max}|x_{0}^{\prime }(k)-x_{i}^{\prime }(k)|$. 0 < ρ < 1 is the discrimination coefficient. The smaller ρ value indicates better discriminant resolution. Generally, ρ = 0.5463 (Deng, 1990). The value of ρ has no effect on the gray relational degree orders of comparative sequences on the reference sequence.

The gray relational degree γ (*x*_0_, *x*_i_) between the reference sequence *x*_0_ and the comparative sequence *x*_i_, was the average of the gray relevancy coefficient values, shown as followed. Because of any two sequence cannot be strictly independent in the gray system, the range is 0 < γ(*x*_0_, *x*_i_) ≤ 1.

(3) $$\gamma (x_{0},x_{i})=\frac{1}{n}\sum_{k}^{n}\xi_{0i}(k)\;(k=1,2,...,n;\;i=1,2,...,m)$$

In this study, the reference sequence *x*_0_ was peak pullout resistance; the comparison sequences *x*_1_, *x*_2_, and *x*_3_ represented root diameter, root length and root surface area, respectively.

### Data Analysis

Statistical analysis of the data was performed using SPSS 16.0 for Windows software (SPSS, Chicago, IL, USA). The relationship between different variables was analyzed by power law function. The difference of root morphological indices and peak pull force between VPT test and OPT test was tested by independent sample *T* test. Figures were drawn by Excel 2007 (Microsoft Corporation).

## Results

### Root Morphological Indices

Root diameter of VPT test and OPT test was 0.30–1.64 mm, and 0.42–1.56 mm, respectively, with an average value of 0.94 ± 0.08 mm, and 0.97 ± 0.07 mm (mean ± SE). Root length was 163.94 ± 14.25 mm (VPT) and 226.80 ± 20.94 mm (OPT), and root surface area was 272.27 ± 43.75 mm^2^ (VPT) and 382.38 ± 55.89 mm^2^ (OPT). Difference of root diameter was insignificant, whereas differences of root length and surface area were significant, between the two tests (Table 1).

**Table 1:** Root morphological index and peak pullout force of vertical pullout test (VPT) and 45° oblique pullout test (OPT) of alfalfa roots.

**Pullout test**		**Root morphological index**			**Peak pullout force/*N***
		**Root diameter/mm**	**Root length/mm**	**Root surface area/mm^2^**	
VPT	Range	0.30–1.64	61.76–334.90	29.09–862.30	4.0–38.8
	Mean ± SE	0.94 ± 0.08^a^	163.94 ± 14.25^a^	272.27 ± 43.75^a^	17.2 ± 2.3^a^
OPT	Range	0.42–1.56	80.09–342.90	52.95–841.74	9.0–60.4
	Mean ±SE	0.97 ± 0.07^a^	226.80 ± 20.94^b^	382.38 ± 55.89^b^	28.2 ± 3.8^b^

*SE is standard error. Different lowercase letters in the columns show significant difference between VPT and OPT at level P < 0.05*.

### Peak Pullout Resistance

**Figure 3:**
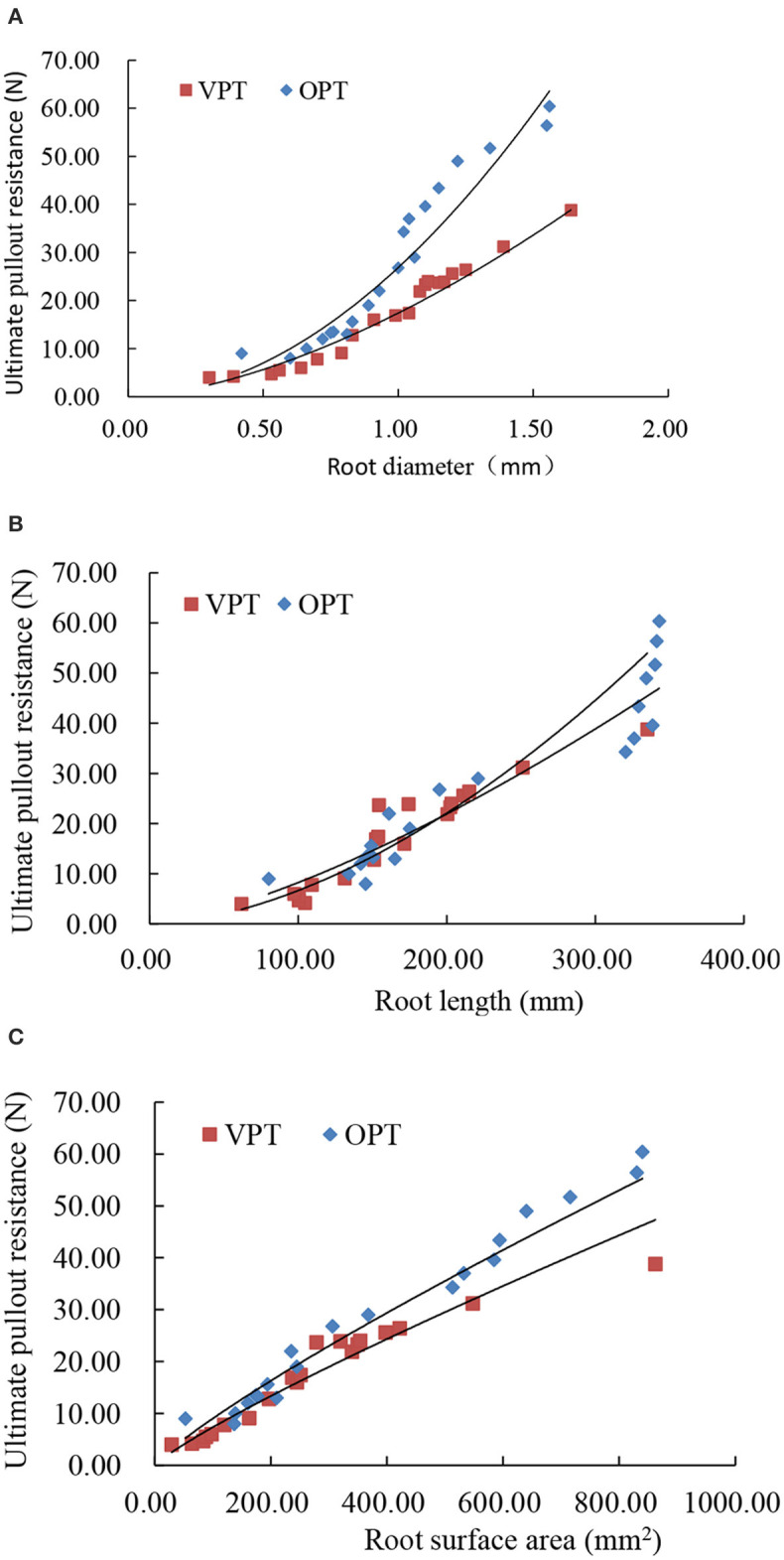
Relationship between root diameter **(A)**, root length **(B)**, and root surface area **(C)** and peak pullout resistance of alfalfa roots under vertical pullout test (VPT) and 45° oblique pullout test (OPT).

The peak pullout resistance of VPT test and OPT test was 4.0–38.8 and 9.0–60.4 N, with an average value of 17.2 ± 2.3 and 28.2 ± 3.8 N (Table 1). The peak pullout resistance of OPT test was significantly greater than that of VPT test. The peak pullout resistance increased with root diameter, root length and root surface area (Figure 3) in power law functions (Table 2).

**Table 2:** Results of regression analysis and relational degree between peak pullout force (*F*) and root size indices for vertical pullout test (VPT) and 45° oblique pullout test (OPT) of alfalfa roots.

**Root morphological index**	**VPT**			**OPT**		
	**Function**	***R*^**2**^**	**γ**	**Function**	***R*^**2**^**	**γ**
Root diameter (*D*)	*F* = 17.397*D*^1.624^	0.932	0.720	*F* = 26.862*D*^1.937^	0.891	0.581
Root length (*L*)	*F* = 0.012*L*^1.413^	0.885	0.711	*F* = 0.002*L*^1.732^	0.88	0.686
Root surface area (*S*)	*F* = 0.137*S*^0.865^	0.940	0.850	*F* = 0.179*S*^0.851^	0.921	0.858

*R^2^ is the coefficient of determination; γ is the gray relational degree*.

### Gray Relational Analysis

The correlation between the three root morphological indices, root diameter, root length and root surface area and the peak root pullout resistance was obtained by the gray relational analysis. Root surface area was the most closely correlated to peak root pullout resistance, and the maximum gray relational degree was 0.850 in VPT test, and 0.858 in OPT test (Table 2), respectively, indicating that root surface area had a relatively more significant effect on peak pullout resistance of the alfalfa roots among the three root morphological indices. The minimum gray relational degree was 0.711 between root length and peak pullout resistance for VPT test, and 0.581 between root diameter and root pullout strength for OPT test, respectively, indicating that root length for VPT test and root diameter for OPT test were root morphological indices having low correlation with peak pullout resistance.

## Discussion

### Root Pullout Properties

Two failure modes may be existed in root pullout test, pullout roots, and breaking roots (Giadrossich et al., 2017). When a root is pulled, it would be pulled out of the soil if the force required to break the root–soil friction bond is less than the force required to break the root, otherwise the root would be broken (Pollen, 2007). Alfalfa roots studied in the VPT and OPT tests were pulled out, and no roots was broken. The first reason was that alfalfa roots grown for 12 months in this study were tap roots, lacking in lateral resistance by branches. The second reason was that the soil moisture content during the tests was relatively high, which resulted in weak friction, as the strength of frictional bonds between the roots and soil, determining the root pullout resistance, was largely dependent of soil shear strength and soil moisture (Pollen, 2007; Fan and Su, 2008; Schwarz et al., 2010).

A power law relationship was observed between the peak pullout resistance and the diameter of alfalfa roots. The thicker roots had larger peak pullout resistance, which is consistent with the previous researches (Pollen, 2007). Usually, the pullout resistance increases with root length (Ennos, 1990). This is one of the reasons why people usually choose long-rooted vegetation, such as alfalfa, black locust, David peach, and sea buckthorn, for soil conservation in loess area (Fang et al., 2016). Power function between peak pullout resistance and root surface area in this study is different from the linear relationship obtained by Hu et al. (2012). The possible reason could be attributed to different soil types and plant types used in the different studies. Different soil types results in different frictions and different pullout resistance (Dupuy et al., 2005). Diverse plants have different contents of internal chemical components, cellulose, lignin, hemicellulose, etc. in roots, which causes a large difference in mechanical properties of roots (Genet et al., 2005; Zhang et al., 2014).

### Effects of Root Morphological Indices on Root Pullout Resistance

A major limitation of the root morphological index for root pullout resistance is that root diameter and length might be overvalued than root surface area in terms of characterizing root pullout resistance, and therefore root surface area is omitted in root reinforcement model (Schwarz et al., 2010). For example, in Mickovski et al. (2007)'s study, the greater pullout resistance of wooden roots than rubber roots was partly attributed to root diameter, as the former was 29% larger in diameter than the later. Stokes et al. (1996) stated that one of the dominant factors influencing pullout resistance of roots was the length of roots in the soil. In this study, root diameters cannot be used to indicate the significant difference of root pullout resistance between the VPT test and OPT test because they were not significantly different, but root lengths and root surface areas could be used. The relational degree is a statistical index which can show the degree of correlation between the peak pullout resistance of roots and root morphological indices. The larger the value of relational degree, the greater the contribution of root morphological indices to the peak pullout resistance of roots. Based on the results of gray relational degree, root surface area had the closest correlation with peak pullout force in the two pullout tests among the three root morphological indices, root diameter, root length, and root surface area, suggesting that root surface area is more suitable for characterizing the difference in the peak pullout resistance of roots. Besides, the coefficient of determination *R*^2^ (Table 2), which was 0.940 in the VPT test and 0.921 in the OPT test, also indicated that the best fit of the regression curves was between root surface area and pullout resistance. Large root surface area provides great bonding area between roots and soil particles, and friction at the interface is more sufficient, so that the roots are capable of resisting more friction and performing greater peak pullout resistance. The important role of root surface area was identical to the previous research results, for example, finer roots having better pullout resistance than their larger counterparts at equivalent root area ratios (RAR) was due to their higher specific surface areas (Gray and Sotir, 1996). It should be noticed that root geometry is another factor relevant to root surface area affecting root peak pullout resistance, because different shaped roots would result in different root pullout resistance although root surface areas are similar. For example, tap- and heart-shaped roots could be more favorable in resisting pullout than plate-shaped roots (Mickovski et al., 2010; Kamchoom et al., 2014). However, existed researches showed that the effect of plant root type on root pullout resistance is mainly reflected in root surface area. Chang et al. (2018) divided *Photinia fraseri* root system into the Y-type, the horizontal root system and the mail direct root system. With the increase of root surface area, the pullout parameters of the three kinds of roots increased in the form of logarithm or power function, and the increasing trend was almost the same. Mickovski et al. (2007) studied the pullout characteristics of three types of root analogs, tap root, herringbone, and dichotomous in sandy soil. The analogs with lateral roots embedded more deeply showed the greatest resistance. In other words, generally, the larger root surface area can result in greater friction and higher pullout force of roots.

### Effects of Pullout Direction on Root Pullout Resistance

Root pullout resistance, a mechanical parameter of roots, is integrated into root cohesion models, fiber bundle model (FBM) (Pollen and Simon, 2005) and root bundle model (RBM) (Schwarz et al., 2013), to evaluate the ability of soil reinforcement by roots. Forty-five degree oblique pulling in our study resulted in significantly greater root pullout resistance than vertical pulling. Tosi (2007) also implicated that the angle between pullout force and trench wall, i.e., pulling direction, affected root pullout results. The maximum root pullout resistance could be achieved when pulling force is vertical to the lateral roots as suggested by Stokes et al. (1996). Pulling direction of existed *in-situ* root pullout tests is conventionally near horizontal or vertical to a trenched wall (Abernethy and Rutherfurd, 2001; Schwarz et al., 2010). In reality, roots could be oriented various angles to the trenched wall (Waldron, 1977). However, roots respond to gravitational force through directional growth along the gravity vector, also called root gravitropism (Feldman, 1985). Our results suggest that root pullout resistance under vertical pulling test is a good choice for evaluating root reinforcement, because roots are in the minimum resistance status. The vertical pulling can give a safe margin for the influence of plant roots on the safety factor of slope stability.

## Conclusion

To investigate the effects of root morphology and pulling direction on root pullout resistance, vertical pullout test and 45° oblique pullout test of alfalfa (*Medicago sativa* L.) roots were conducted. The results showed that root surface area was a more suitable morphological index for characterizing the peak pullout resistance of different roots than root diameter and root length. It is suggested that if roots are grown downward in regular gravitropism, vertical pullout test should be used to get root pullout resistance, which could give a safe margin for the estimate of slope stability.

## Data Availability Statement

The raw data supporting the conclusions of this article will be made available by the authors, without undue reservation.

## Author Contributions

CZ planned and designed the research. QY and CZ wrote the manuscript. PL performed experiments. JJ analyzed data. All authors have read and approved the final version of the manuscript.

## Conflict of Interest

The authors declare that the research was conducted in the absence of any commercial or financial relationships that could be construed as a potential conflict of interest.
